# Prevalence of Radiographic Osteoarthritis of the Knee and Its Relationship to Self-Reported Pain

**DOI:** 10.1371/journal.pone.0094563

**Published:** 2014-04-10

**Authors:** Lan T. Ho-Pham, Thai Q. Lai, Linh D. Mai, Minh C. Doan, Hoa N. Pham, Tuan V. Nguyen

**Affiliations:** 1 Division of Bone and Muscle Research, Faculty of Applied Sciences, Ton Duc Thang University, Ho Chi Minh City, Vietnam; 2 Pham Ngoc Thach University of Medicine, Ho Chi Minh City, Vietnam; 3 Department of Rheumatology, People's Hospital 115, Ho Chi Minh City, Vietnam; 4 Osteoporosis and Bone Biology Program, Garvan Institute of Medical Research, Sydney, Australia; 5 School of Public Health and Community Medicine, University of New South Wales, Sydney, Australia; 6 Centre for Health Technologies, University of Technology, Sydney, Australia; University of South Australia, Australia

## Abstract

**Background and Aim:**

Osteoarthritis (OA) of the knee is one of the most common skeletal disorders, yet little data are available in Asian populations. We sought to assess the prevalence and pattern of radiographic OA of the knee, and its relationship to self-reported pain in a Vietnamese population.

**Methods:**

The study was based on a sample of 170 men and 488 women aged ≥40 years who were randomly sampled from the Ho Chi Minh City (Vietnam). Radiographs of the knee were graded from 0 to 4 according to the Kellgren and Lawrence scale. Osteoarthritis was defined as being present in a knee if radiographic grades of 2 or higher were detected. Knee pain and symptoms were ascertained by direct interview using a structured questionnaire.

**Results:**

The point prevalence of radiographic OA of the knee was 34.2%, with women having higher rate than men (35.3% vs 31.2%). The prevalence of knee OA increased with advancing age: 8% among those aged 40–49 years, 30% in those aged 50–59 years, and 61.1% in those aged ≥60 years. Greater BMI was associated with higher risk of knee OA. Self-reported knee pain was found in 35% of men and 62% of women. There was a statistically significant association between self-reported knee pain and knee OA (prevalence ratio 3.1; 95% CI 2.0 to 4.6).

**Conclusions:**

These data indicate that approximately a third of Vietnamese men and women have radiographic OA in the knee, and that self-reported knee pain may be used as an indicator of knee osteoarthritis.

## Introduction

Osteoarthritis (OA), a degenerative joint disease, is a major public health problem in the general population because it is highly prevalent among the elderly and is associated with considerable disability. Symptomatic OA is less common than asymptomatic (radiographic) OA. In Caucasian populations, the prevalence of symptomatic OA is around 10% in men and 20% in women aged 45 years and above [1], but for radiographic OA, the prevalence could be between 27% and 80% [2]. OA can affect multiple joints in the body, but it is commonly found in the knee. A recent analysis of data from the National Health and Nutrition Examination Survey III (NHANES III) found that approximately 35% of women and men aged 60 years and above had radiographic OA of the knee [3]. Moreover, OA is recognized as the most common cause of disability in the elderly [4], with approximately 85% of all knee and hip replacements being due to OA [5].

OA is a major cause of musculoskeletal pain/complaints. Studies in European populations have reported that the prevalence of musculoskeletal pain ranged between 30% and 80% [6]–[8]. Knee pain is strongly associated with knee OA. Indeed, a study in 819 individuals aged ≥50 years found that among those who self-reported knee pain, knee OA was found in 77% of men and 61% of women [9]. However, it is not entirely clear whether self-reported symptoms and knee pain can be used as an indicator of knee OA.

Although risk factors for prevalence of OA have been well studied in Caucasian populations, little data are available in Asian populations. Asia is where the aging of population will impose a significant skeletal burden in the future. The proportion of people aged 65 years and older in Asia is estimated to increase from ∼7% in 2008 to 16% in 2040 [10]. In a recent review, it is concluded that the prevalence knee OA or knee pain is as high as or higher than other Caucasian populations [11]. For instance, in a Chinese population aged ≥60 years, the prevalence of knee OA was 22% in men and 43% in women, and this prevalence was 45% higher than that in US White population [12]. In a Japanese rural population, the prevalence of knee OA was 30% in women and 11% in men [13]. In Vietnam, a country with a population of 90 million, the prevalence of OA has not been studied, but the prevalence of knee pain was 18% among people aged 16 and above [14]. However, data on the prevalence of radiographic OA of the knee is not available in many Asian populations. In an effort to ascertain the magnitude of OA in the Asian population and to contribute to the international literature of OA, we have undertaken a study to estimate the prevalence and to examine the pattern of radiographic OA of the knee in a Vietnamese population.

## Study Design and Methods

### Study design

The study was designed as a cross-sectional investigation, with the setting being Ho Chi Minh City, a major city in Vietnam. The study was conducted in accordance with the principles of medical ethics of the World Health Organization. All participants were provided with full information about the study's purposes, and gave written informed consent to participate in the study. The research protocol and procedures were approved by the Scientific Committee of the People's Hospital 115 and Pham Ngoc Thach University of Medicine.

We approached community organizations, including church and temples, and obtained the list of members aged 18 years and above. In the next step, we used a computer program to randomly select individuals in the list. We used simple random sampling technique to identify potential participants. We sent a letter of invitation to the selected individuals. Some participants (approximately 5%) did not respond to our letter of invitation, and we contacted them via phone. The participants did not receive any financial incentive, but they received a free health check-up, and lipid analyses. Participants were excluded from the study if they had rheumatoid arthritis. In this report, we included participants aged 40 years and older because OA of the knee mainly affects people in that age range [15].

### Measurements

All participants underwent a detailed investigation to obtain the following baseline data: a standardized interview gathered information on demographic data, lifestyle, and nutritional status. Anthropometric parameters including age, weight, standing height were obtained. Body weight was measured on an electronic scale with indoor clothing without shoes. Height was determined without shoes on a portable stadiometer with mandible plane parallel to the floor. Body mass index (BMI) was calculated as weight in kg over height in meter squared.

Each participant was asked to provide information on current and past smoking habits. Smoking was quantified in terms of the number of pack-years consumed in each ten-year interval age group. Alcohol intake in average numbers of standard drinks per day, at present as well as within the last 5 years, was obtained. Clinical data including blood pressure, pulse, and reproductive history (i.e. parity, age of menarche, and age of menopause), medical history (i.e. previous fracture, previous and current use of pharmacological therapies) were also obtained. The interview was conducted by the research team which included doctors and nurses who were completely blind to radiographs.

Knee pain and clinical symptoms were assessed by the KNEST questionnaire [16] and the American College of Rheumatology criteria for the classification and reporting of osteoarthritis of the knee [17] which have been modified for the Vietnamese population. The questionnaire consists of 9 questions with dichotomous answer (yes/no) as follows: knee pain including pain during movement, pain when going upstairs, pain when squatting, pain at rest; stiffness <30 minutes; crepitus; bony tenderness, bony enlargement, and deformity. The presence of any kind of knee pain and any of the clinical symptoms for at least 3 months over the past 12 months was considered having knee pain or symptom. The reliability of the questionnaire was tested in a random sample of 30 individuals, and the coefficient of reliability was consistently greater than 0.80.

### Radiographic assessment

The ascertainment of OA was based on radiographic assessment using the Kellgren - Lawrence scoring system which is recommended by the WHO as a standard method for studying OA in epidemiologic studies [18]. Anterior-posterior radiographs of both knees were taken from all participants. Radiographs were read by a single rheumatologist (LHP) who had more than 12 years experience in rheumatology practice. The rheumatologist was completely unaware of the clinical conditions of participants. In each knee, the presence or absence of osteophytes, joint space narrowing, sclerosis and cysts was examined for each hand joint using the Kellgren-Lawrence system of scoring: 0 =  none, 1 =  possible osteophytes only, 2 =  definite osteophytes and possible joint space narrowing, and 4 =  large osteophytes, severe joint space narrowing, and/or bony sclerosis. The presence of radiographic OA was defined if the grade was 2 or more in at least one joint.

### Data analysis

The analysis plan was initiated prior to the data collection and ascertainment of OA of the knee. In the descriptive analysis, we determined the point prevalence of radiographic OA in the knee by age group and BMI group. We grouped participants into three 10-year age groups: under 50, 50 to 59, and 60 years or above. BMI was classified into 4 groups according to the classification of obesity for Asians: under 18.5, 18.5 to 22.9, 23.0 to 24.9, and 25 or above. In the next stage, we modeled the risk of OA as a function of gender, age, BMI, and OA pain/symptom. The primary model of analysis was the negative binomial regression model [19], which is considered appropriate for outcomes with high prevalence, as it avoids the problem of exaggerated odds ratio in the logistic regression model. Both univariate and multivariate regression models were used to estimate the magnitude of association between knee OA and potential risk factors. In the univariate analysis, each predictor (or risk factor) was analyzed separately, without adjustment for any other factor in the model. In the multivariate analysis, all risk factors were analyzed simultaneously in a model. Because the risk factors could be correlated with each other, we searched for a set of independent risk factors using the Akaike Information Criterion (AIC) based method. Based on the AIC values, we selected the “best” model, and using parameter estimates of this model, we calculated the prevalence ratio (i.e., ratio of the prevalence of OA among those with exposure to a risk factor over the prevalence among those without exposure to the risk factor). All analyses were conducted using the R Statistical Environment [20].

## Results

The study involved 170 men and 488 women, whose demographic and lifestyle characteristics are shown in **Table 1**. The individuals' ages ranged between 40 and 98, with average age being 55.5 years. More than a-third of men and women aged 60 years and above. Approximately 21% of the women and 32% of the men had BMI greater than 25 kg/m^2^ which are considered “obese” by Asian criteria. As expected, the prevalence of cigarette smoking and alcohol use in women was very low (less than 1%) compared with men (56%). The proportion of individuals who had completed secondary school education was greater in women (75%) than in men (64%); however, men were more than twice as likely as women to have tertiary education (24% vs 11%).

**Table 1:** Characteristics of 488 women and 170 men in the study.

Characteristics	Women (n = 488)	Men (n = 170)	P-value
N	488	170	
Age	55.9 (12.6)	55.1 (15.8)	0.512
40–49 (n; %)	146 (29.9)	56 (32.9)	
50–59	176 (36.1)	51 (30.0)	
60+	166 (34.0)	63 (37.1)	
Height (cm)	153.2 (5.2)	163.6 (5.7)	<0.0001
Weight (kg)	53.1 (7.5)	62.2 (9.1)	<0.0001
Body mass index (kg/m^2^)	22.6 (2.9)	23.2 (3.2)	0.011
<18.5 (n; %)	24 (4.9)	11 (6.5)	
18.5 to 22.9	256 (52.6)	70 (41.2)	
23.0 to 24.9	106 (21.8)	34 (20.0)	
>25.0	101 (20.7)	55 (32.4)	
**Education attainment (n; %)**			<0.0001
Illiterate	15 (31)	1 (0.6)	
Primary school	85 (17.5)	8 (4.7)	
Secondary school	307 (75.0)	108 (63.9)	
College and university	55 (11.3)	41 (24.3)	
**Occupation (n; %)**			<0.0001
Sales/small business	79 (16.2)	28 (16.6)	
Factory workers	9 (1.9)	9 (5.3)	
Office workers	77 (15.8)	18 (10.7)	
Professionals	12 (2.5)	16 (9.5)	
Retired	69 (14.2)	49 (29.0)	
Housewives	182 (37.4)	-	
Current alcohol drinker (n; %)	14 (2.9)	102 (60.4)	<0.0001
Current smoking (n; %)	6 (1.2)	94 (55.6)	<0.0001
Current tea drinker (n; %)	249 (51.1)	98 (58.0)	<0.0001

### Prevalence of knee osteoarthritis

The overall prevalence of radiographic knee OA was 34.2% (n = 225), with women having higher prevalence than men (35.3% vs. 31.2%; **Table 2**). A majority of knee OA was in the form of osteophytes (32.3% in women and 25.3% in men). Joint space narrowing was found in 24% of women and 19% of men. There was no significant difference in the prevalence of knee OA between left knee and right knee.

**Table 2:** Prevalence of osteoarthritis of the knee in 170 men and 488 women.

Radiographic OA	Women (n; %)	Men (n; %)	P-value
**Joint space narrowing**	**115 (23.6)**	**33 (19.4)**	0.263
Right knee	112 (22.9)	31 (18.2)	0.200
Left knee	108 (22.1_	32 (19.8)	0.364
**Osteophytes**	**158 (32.4)**	**43 (25.3)**	0.084
Right knee	148 (30.3)	43 (25.3)	0.213
Left knee	144 (29.5)	40 (23.5)	0.135
**Joint space narrowing or osteophytes**	**172 (35.3)**	**53 (31.2)**	0.337
Right knee	165 (33.8)	53 (31.2)	0.530
Left knee	164 (33.6)	51 (30.0)	0.388
Knee OA	172 (35.3)	53 (31.2)	0.335

**Notes:** Numbers shown in the table are actual number of individuals and percent of sex-specific total (in bracket).

**Figure 1:**
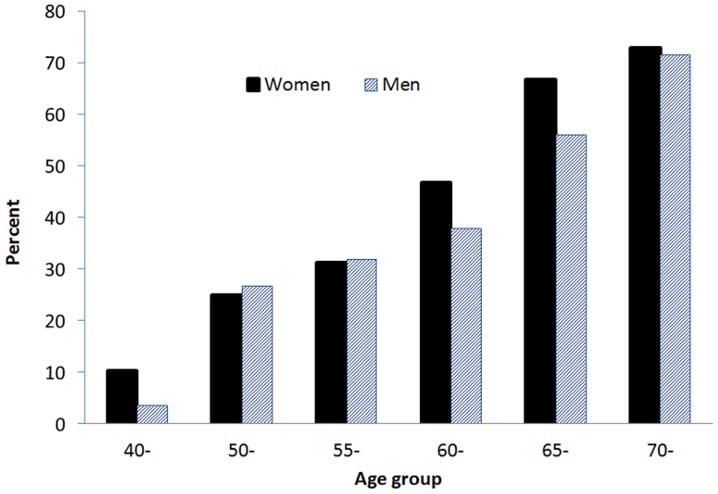
Prevalence of osteoarthritis of the knee in 170 men and 488 women aged ≥40 years classified by age.

**Figure 2:**
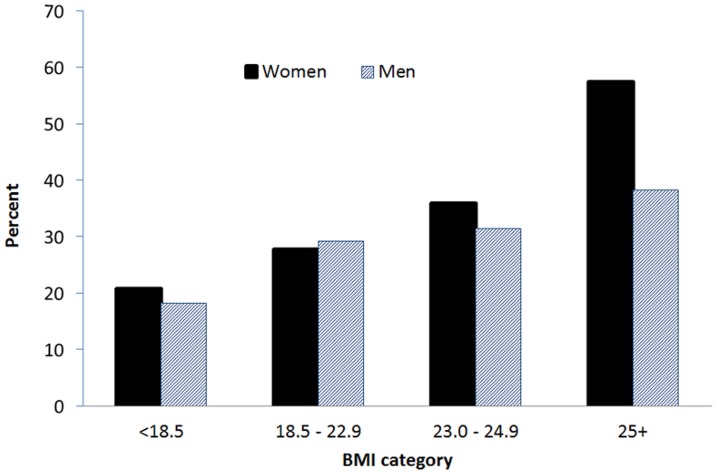
Prevalence of osteoarthritis of the knee classified by body mass index group in 170 men and 488 women aged ≥40 years.

Advancing age was associated with an increased risk of radiographic OA of the knee (**Figure 1**). Among those aged between 40 and 49, approximately 8.5% had knee OA, and this prevalence rate increased to 30% in those aged between 50 and 59, and 61% in those aged ≥60 years. Furthermore, greater BMI was associated with a greater risk of OA, such that obese individuals had higher prevalence of knee OA than non-obese individuals. For instance, the prevalence of knee OA among those with BMI ≥25 was 47.4%, two-fold higher than the risk among those with BMI <18.5 kg/m^2^ (**Figure 2**).

### Self-reported pain and radiographic OA

The prevalence of self-reported pain and symptoms in the knee was 55% (n = 363), with women reporting more frequent pain or symptoms (62%, n = 304) than men (35%, n = 59) (**Table 3**). A majority of the self-reported complaint was pain when going upstairs (42% of women and 20% of men), pain when squatting (40% of women and 11% of men), and crepitus (38% of women and 17% of men). Bony tenderness was reported in only 2.3% of women and ∼1% of men.

**Table 3:** Self-reported pain and clinical symptoms of the knee in 170 men and 488 women.

	Women (n; %)	Men (n; %)	P-value
Pain during movement	185 (37.9)	27 (15.9)	<0.0001
Pain when going upstairs	204 (41.8)	34 (20.0)	<0.0001
Pain when squatting	196 (40.2)	19 (11.2)	<0.0001
Pain at rest	43 (8.8)	8 (4.7)	0.084
Bony tenderness	11 (2.3)	1 (0.6)	0.162
Bony enlargement	40 (8.2)	4 (2.4)	0.009
Crepitus	186 (38.1)	29 (17.1)	<0.0001
Stiffness < 30 minutes	86 (17.6)	12 (7.1)	0.009
Deformity	21 (4.3)	3 (1.8)	0.128
Any symptom/pain	304 (62.3)	59 (34.7)	0.0001

**Notes:** Numbers shown in the table are actual number of individuals and percent of sex-specific total (in bracket).

In the multivariable negative binomial regression analysis, advancing age and greater BMI were independently associated with a greater risk of OA of the knee (**Table 4**). Each 5-year increase in age was associated with 56% increase (PR 1.56, 95% CI 1.43 to 1.71) in the risk of knee OA. Moreover, each kg/cm^2^ increase in BMI was associated with a 14% increase (PR 1.14, 95% CI 1.07 to 1.23) in the risk of OA of the knee. Apart from age and BMI, 3 knee complaints were also independently associated with increased risk of knee OA: pain when squatting (PR 2.19; 95% CI 1.42–3.39), bony enlargement (PR 3.54; 95% CI 1.57–8.01), and crepitus (PR 1.81; 95% CI 1.18–2.79). The area under the receiver operating characteristic curve (ROC) was 0.83. Gender was not an independent predictor of knee OA when the above factors were considered in the model.

**Table 4:** Predictors of radiographic OA of the knee: results of binomial regression analysis on 170 men and 488 women.

Factor	Prevalence ratio and 95% CI	P-value
**Model I**		
Gender (women)	1.15 (0.71–1.85)	0.565
Age (+5 yr)	1.56 (1.43–1.71)	<0.0001
BMI (+1)	1.14 (1.07–1.23)	0.0001
Pain when squatting (Yes)	2.19 (1.42–3.39)	0.0004
Bony enlargement (Yes)	3.54 (1.57–8.01)	0.002
Crepitus (Yes)	1.81 (1.18–2.79)	0.007
**Model II**		
Gender (women)	1.12 (0.70–1.79)	0.417
Age (+5 yr)	1.54 (1.41–1.69)	<0.0001
BMI (+1)	1.14 (1.06–1.22)	0.0002
Pain when squatting (Yes)	1.41 (1.27–1.57)	<0.0001

**Figure 3:**
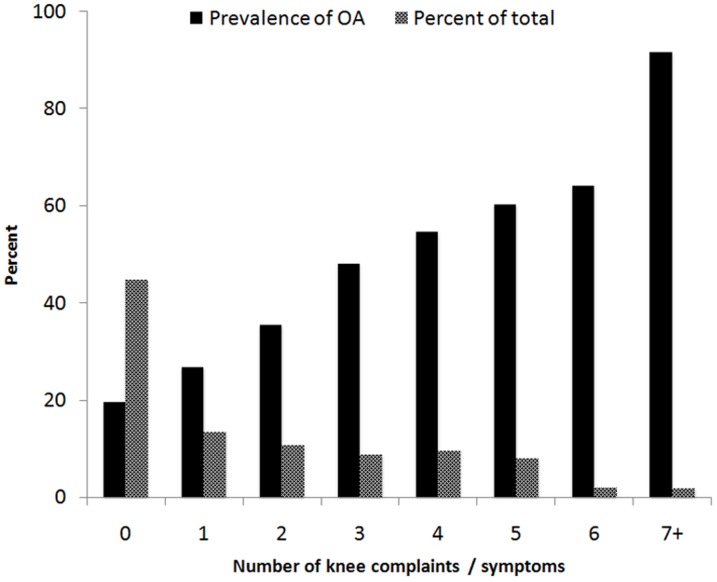
Prevalence of osteoarthritis of the knee classified by the number of knee complaints or symptoms among 170 men and 488 women aged ≥40 years. The solid bar shows the prevalence of knee OA, and the cross-hatched bar shows the proportion of individuals with 0, 1, 2, …, 7+ knee complains/symptoms.

In a further analysis, we sum the number of individual knee complaints (e.g., pain and symptoms) to obtain an overall score which ranged between 0 and 9. Individuals with higher score had greater risk of OA of the knee. For example, among those reporting only 1 complaint/symptom, 27% had knee OA; this proportion increased to about 50% among those with 3 complaints/symptoms, and 92% among those with 7 or more complaints/symptoms (**Figure 3**). However, it is interesting to note that among those reporting no symptom or no pain at all, radiographic of the knee was found in 20% of them. The score was then considered in the multivariable negative binomial regression model. After adjusting for age and BMI, each score increase was associated with a 41% increase in the risk of knee OA (PR 1.41; 95% CI 1.27 to 1.57). The area under the ROC curve for the model with age, BMI and pain score was 0.83.

## Discussion

Osteoarthritis of the knee is a significant public health problem in the general population, because it is associated with a substantial disability and healthcare costs. However, the prevalence of and risk factors for knee OA have not been well documented in Asian populations. In this population-based study in a Vietnamese population, we have shown that the prevalence of radiographic knee OA is as high as in Caucasian populations. We also found that self-reported knee pain and complaints were strongly associated with the risk of knee OA, but the magnitude of association is not high enough for a reliable discrimination between those who have and those who do not have radiographic knee OA. These findings deserve further elaboration.

In this study, approximately 34% of individuals had radiographic OA of the knee. As observed in most previous studies [9], [12], [21]–[26], we also found that women have higher prevalence of radiographic knee OA than men. Our estimated prevalence rate is comparable with the prevalence in White US population [3], but lower than the Chinese (43%) [12] and Japanese populations [25]. Since all studies used the Kellgren-Lawrence method of diagnosis, the difference is probably due to population characteristics and sampling variability. In our study, the minimum age was 40 years, whereas in studies by Zhang et al [12] and Muraki et al [25], the minimum age was 60 years.

We found a statistically significant but modest association between age, BMI and radiographic knee OA. This association has also been observed in several previous studies. Obesity is one of the well documented risk factors for OA of the knee [27], probably mediated through adipokines [28] which has been shown to promote chronic low-grade inflammatory state in joints [29], [30]. However, in our population, only 2% of men and women had BMI ≥30 kg/m^2^, and this probably explains the modest association between BMI and knee OA in the present study. As observed in previous studies [2], [23]–[26], [31]–[34], we also found that the prevalence of OA of the knee increased with advancing age, and the increase appeared to be more pronounced in women than in men. Although it has been suggested that the gender-related difference in OA could be due to the higher prevalence of obesity in women than in men [35], the difference in obesity could not explain the gender difference in knee OA in the present study where men actually had higher prevalence of obesity than women.

Our data show that the prevalence of self-reported knee complaints or pain is high. Indeed, 62% of women and 35% of men reported to suffer from knee pain and/or knee symptoms. A recent German study reported that 63% of women and 57% of men aged ≥40 years reported to have pain and/or joint complaints [15]; these figures are comparable with our estimates. In a Japanese population based study, knee pain was found in 38% of women and 24% of men [25]. Taken together, self-reported knee pain and complaints are high in the general community.

Interestingly, we found that self-reported knee complaints are highly associated with knee OA. Among the complaints, we identified 3 major predictors of radiographic knee OA: pain when squatting, bony enlargement and crepitus. In Vietnam, people have the habit of squatting or sitting on the floor (which is uncommon in Western countries), and it has been postulated that squatting may in fact protect against OA of the hip [36], and help reduce knee OA. Thus, a pain when squatting appeared to be a good signal of knee OA. Knee pain or knee complaint can be originated from different causes, including inflammatory arthritis, sepsis, injury, periarticular tendonopathy, and knee OA. Thus, a knee pain or knee complaint is not necessarily a specific marker of knee OA, and a discordance between knee pain or knee complaint and radiographic OA of the knee [37] is expected. However, we found that the number of knee complaints/symptoms, particularly bony enlargement and crepitus, when combined age and BMI, can be a good predictor of knee OA.

The present results have to be interpreted within the context of strengths and potential limitations. The study was based on a reasonably large sample size, and the participants were randomly selected using a rigorous random sampling technique to ensure the representativeness of the general population. The study population is highly homogeneous, which reduces the effects of potential confounders that could compromise the estimates. Nevertheless, the participants in this study were sampled from an urban population; as a result, the study's finding may not be generalizable to the rural populations. Because we excluded individuals with diseases deemed to interfere with bone metabolism, the prevalence of knee OA reported here could be an underestimate of the true prevalence in the general population. Moreover, the Kellgren-Lawrence method may not be as sensitive and accurate as the MRI technique in the diagnosis of knee OA. The questionnaire for knee pain/symptoms used a dichotomous scale which did not allow us to quantify the severity of pain, and hence could limit the analysis of association between knee pain and knee OA.

In summary, we found that the prevalence of radiographic OA of the knee in this Vietnamese urban population is approximately 34%, with women having a higher prevalence than men. We also demonstrated that advancing age, greater body mass index, and self-reported knee pain and/or symptoms are associated with a greater risk of radiographic OA of the knee. Given the rapid aging population in Asia, these findings suggest that osteoarthritis of the knee will become a significant public problem in Asian populations.
